# LazyFox: fast and parallelized overlapping community detection in large graphs

**DOI:** 10.7717/peerj-cs.1291

**Published:** 2023-04-20

**Authors:** Tim Garrels, Athar Khodabakhsh, Bernhard Y. Renard, Katharina Baum

**Affiliations:** 1 Hasso Plattner Institute for Digital Engineering gGmbH, Potsdam, Germany; 2 Digital Engineering Faculty, University of Potsdam, Potsdam, Germany; 3 Department of Mathematics and Computer Science, Free University Berlin, Berlin, Germany; 4 Windreich Department of Artificial Intelligence and Human Health, Icahn School of Medicine at Mount Sinai, New York, USA; 5 Hasso Plattner Institute for Digital Health at Mount Sinai, Icahn School of Medicine at Mount Sinai, New York, USA

**Keywords:** Overlapping community detection, Large networks, Weighted clustering coefficient, Heuristic triangle estimation, Parallelized algorithm, C++ tool, Runtime improvement, Open source, Graph algorithm, Community analysis

## Abstract

The detection of communities in graph datasets provides insight about a graph’s underlying structure and is an important tool for various domains such as social sciences, marketing, traffic forecast, and drug discovery. While most existing algorithms provide fast approaches for community detection, their results usually contain strictly separated communities. However, most datasets would semantically allow for or even require overlapping communities that can only be determined at much higher computational cost. We build on an efficient algorithm, Fox, that detects such overlapping communities. Fox measures the closeness of a node to a community by approximating the count of triangles which that node forms with that community. We propose LazyFox, a multi-threaded adaptation of the Fox algorithm, which provides even faster detection without an impact on community quality. This allows for the analyses of significantly larger and more complex datasets. LazyFox enables overlapping community detection on complex graph datasets with millions of nodes and billions of edges in days instead of weeks. As part of this work, LazyFox’s implementation was published and is available as a tool under an MIT licence at https://github.com/TimGarrels/LazyFox.

## Introduction

Graphs, also called networks, are present in many fields as they capture the interaction of entities as edges between nodes representing those entities. Communities (or clusters, or modules) are considered to be important network structures (Fortunato & Newman, 2022) as they describe functionally similar groups within networks. The identification of such groups is relevant in various domains such as biology (Barabasi & Oltvai, 2004; Regan et al., 2002; Boccaletti et al., 2006; Guimerà & Amaral, 2005; Ahn, Bagrow & Lehmann, 2010), medicine (Barabasi, Gulbahce & Loscalzo, 2011; Gavin et al., 2006) and technical infrastructure (Regan & Barabasi, 2003; Guimerà et al., 2005). In social networks, communities can identify common interest groups or friend circles (Mcauley & Leskovec, 2014). In drug research, new, targetable proteins can be discovered by clustering proteins into functionality groups (Ma et al., 2019). Similar products of an online shop can be grouped together to derive product recommendations (Basuchowdhuri, Shekhawat & Saha, 2014).

While community itself is a term whose precise definition is highly context dependent (Kelley et al., 2012; Fortunato & Newman, 2022), various algorithms have been developed to detect such structures (Fortunato, 2010). In this work, we focus on communities of bond. These are communities where the membership of a community is based on the relation to other members—their connectivity in the network—rather than an affinity to the identity of that group as a whole. This naturally leads to more interconnections between group members (Ren, Kraut & Kiesler, 2007). Algorithms detecting such communities typically exploit this property by optimizing communities so that nodes of one group are more densely connected with each other than with nodes outside of that group.

### Disjoint community detection

Disjoint communities capture structural node clusters within a network where each node belongs to exactly one community. Multiple different algorithms for disjoint community detection have been proposed, that have been categorized into cut-based (minimize edges between communities), cluster-based (communities of bond, maximal connectivities), structure-based (*e.g*., stochastic block models), and dynamic-based (*e.g.*, random walk, diffusion) methods (Schaub et al., 2017). Alternatively, a simpler classification into bottom-up, top-down and structure-based approaches has been suggested (Souravlas et al., 2021). The performance of multiple of these algorithms particularly in terms of community structure on 100 real-world datasets has been compared recently by Dao, Bothorel & Lenca (2020).

One of the most common approaches for community detection is to define a metric that measures desired structural properties of a community (Lancichinetti et al., 2011; Yang & Leskovec, 2012; Newman, 2006; Prat-Pérez et al., 2012). By choosing communities that increase the metric over communities that decrease it, the communities are optimized to display that structural property. This way a clear community definition (groups of nodes that result in the highest value of the metric) is created and detected communities are to some degree controllable in their structure.

### Overlapping community detection

While disjoint communities yield valuable information, most real world datasets contain functional groups that are overlapping (Reid, McDaid & Hurley, 2013; Lancichinetti, Fortunato & Kertész, 2009). People can belong to multiple social circles, proteins can have various biological functions or be target of multiple drugs, and products rarely fall into exactly one category. Therefore, traditional community detection algorithms have been adapted and new algorithms have been proposed to detect overlapping communities (Fortunato, 2010). Comparative studies of these algorithms focusing on different performance estimations have been proposed on synthetic (Xie, Kelley & Szymanski, 2013) as well as real-world networks (Jebabli et al., 2018; Vieira, Xavier & Evsukoff, 2020). However, overlapping community detection is a computationally more expensive problem than regular (disjoint) community detection. Long runtimes of days or even weeks make community detection unfeasible in large datasets, which is especially true for overlapping community detection (Lancichinetti & Fortunato, 2009; Danon et al., 2005). This limits the usability of most algorithms to smaller sized networks with edge counts in the lower millions.

### Enabling large scale community detection

Various approaches can be used to improve runtime and enable the use of (overlapping) community detection algorithms on large datasets. Parallelization can leverage modern multi-core CPUs, and algorithms with independent computation steps can profit from parallelization directly (Liu & Chamberlain, 2018; Lu, Halappanavar & Kalyanaraman, 2015). Other algorithms cannot be parallelized without changes to computation logic, however, small alterations often do not influence the results substantially (Prat-Pérez, Dominguez-Sal & Larriba-Pey, 2014). Such parallelized approaches can also be distributed to a multi-machine computing cluster, enabling the use of even more computational resources (Saltz, Prat-Pérez & Dominguez-Sal, 2015). Finally, algorithms relying on metric optimization can also be improved in runtime by replacing the metric with an estimator, an approximation or a related metric that is less computationally expensive. This reduces computational effort and speeds up the community detection.

#### Weighted community clustering score

The weighted community clustering (*WCC*) score (Prat-Pérez et al., 2012) is a metric that has been successfully used to detect communities (Saltz, Prat-Pérez & Dominguez-Sal, 2015; Song, Bressan & Dobbie, 2015; Prat-Pérez, Dominguez-Sal & Larriba-Pey, 2014). It measures structural closeness by counting the number of triangles a node forms with a community to determine the membership of that node to that community. Thereby, it is closely related to the general concept of clustering coefficients and has multiple areas of application, *e.g*., the analysis of protein-protein interaction networks (Omranian, Angeleska & Nikoloski, 2021; Wang et al., 2021), for systemic risk measure in finance (Cerqueti, Clemente & Grassi, 2021), or in trade networks (Bartesaghi, Clemente & Grassi, 2023). In the ‘Methods’ section, we explain this metric in more detail. Community detection algorithms based on this metric optimize communities to maximize the global *WCC* score by allowing nodes to individually join or leave communities. Such algorithms can yield disjoint or overlapping communities depending on whether nodes are allowed to join multiple communities.

#### Efficient *WCC*-based large scale overlapping community detection

*WCC*-based overlapping community detection approaches also suffer from computational complexity issues when dealing with large scale datasets. Thus, parallelized (Prat-Pérez, Dominguez-Sal & Larriba-Pey, 2014) and distributed (Saltz, Prat-Pérez & Dominguez-Sal, 2015) *WCC* versions have been proposed that allow multiple nodes at the same time to decide for changes to the community partition, so those decisions can be computed on separate threads or machines. Furthermore, computationally less expensive estimations of the *WCC* score have been suggested (Prat-Pérez, Dominguez-Sal & Larriba-Pey, 2014; Lyu et al., 2020) to further improve runtime. Thereby, Lyu et al. (2020) have found that a *WCC*-derived metric, $\widehat {WCC}$, that particularly employs approximations of triangle counts in an algorithm they call Fox yields results very similar to calculating the exact count. While their proposed $\widehat {WCC}$ metric allows Fox to compute overlapping communities on large networks with millions of edges, the implementation of Fox was not published making it hard to reproduce the results or to use them for further applications.

#### LazyFox-parallelized large scale overlapping community detection

We here propose our tool for overlapping community detection, LazyFox, that contains an implementation of the Fox algorithm. In addition, we extend the work of Lyu et al. (2020) and combine it with ideas from Prat-Pérez, Dominguez-Sal & Larriba-Pey (2014) by introducing parallelism to leverage the advantage of modern multiprocessor systems to even further accelerate the algorithm.

We show that LazyFox produces extremely similar results to Fox in a fraction of Fox’s runtime, making it more efficient to work with. This also enables the usage of the approach on large scale graphs like the social media network Friendster with millions of nodes and billions of edges (Leskovec & Krevl, 2014). We analyze the impact of hyper-parameters of the Fox and LazyFox algorithm on real-world examples and compare the performance in terms of community quality with other community detection methods. The C++ implementation of LazyFox (which also includes a Fox implementation) is published as open-source under MIT licence (https://github.com/TimGarrels/LazyFox).

In the following sections we introduce the datasets, the algorithmic details of Fox and LazyFox, and the methods used to evaluate them. We describe the impact of our LazyFox contribution on the runtime and community quality of three datasets of different scales and compare it with three other community detection methods. We include the analysis of a fourth dataset to illustrate the ability of LazyFox to handle large scale datasets in contrast to Fox. Finally, we summarize and discuss our results.

## Related work

### Methods for disjoint community detection

While algorithms based on label propagation (Raghavan, Albert & Kumara, 2007), stochastic blockmodels (Hofman & Wiggins, 2008; Wang & Wong, 1987) and game theory (Bu et al., 2017) for disjoint community detection exist, the most common approach is to define a metric that measures desired structural properties of a community such as modularity or clustering scores. The latter class contains algorithms such as the well-known Louvain method that performs modularity optimization (Blondel et al., 2008), and algorithms relying on clustering scores such as the work introducing the *WCC* (Prat-Pérez et al., 2012). A newer approach that has been proposed for disjoint community detection is for example core expansion in that center nodes of communities are determined and then expanded upon (Choumane, Awada & Harkous, 2020). A community detection method based on path analysis and threaded binary trees has been proposed in Souravlas, Sifaleras & Katsavounis (2019).

### Methods for overlapping community detection

Algorithms which detect overlapping communities can use other approaches than disjoint community detection algorithms such as clique expansion (Lee et al., 2010; Palla et al., 2005), matrix factorization in methods such as BigClam (Yang & Leskovec, 2013), other decompositions (Psorakis et al., 2011; Ding et al., 2016), link clustering or partitioning (Shi et al., 2013; Ahn, Bagrow & Lehmann, 2010; Evans & Lambiotte, 2009; Ponomarenko, Pitsoulis & Shamshetdinov, 2021). Some approaches also propose post-processing steps to create overlapping communities from pre-existing disjoint communities (Chakraborty, 2015).

Moreover, approaches that have been proposed to detect disjoint communities can also be adjusted to directly yield overlapping communities. Label propagation based algorithms have been proposed that can allow nodes to hold multiple labels at the same time, thereby creating overlapping communities (Xie & Szymanski, 2012; Gregory, 2010). Stochastic blockmodels supporting mixed memberships have been developed (Airoldi et al., 2008; Gopalan & Blei, 2013). And if an algorithm is based on the optimization of a metric measuring structural properties, allowing nodes to join multiple communities during the optimization can also change the result from disjoint to overlapping communities (Lancichinetti & Fortunato, 2009), for example optimizing the local statistical significance of communities according to a global null model in the OSLOM algorithm (Lancichinetti et al., 2011).

### *WCC*—clustering and adaptations for community detection

Communities of bond that we focus on in this work benefit from optimizing connectivity between nodes within a community. Such connectivity is expressed in terms of triangles and clustering scores. These components are the core of the *WCC* score (Prat-Pérez et al., 2012).

Adaptations of the *WCC* score have been suggested by Midoun, Wang & Talhaoui (2021) for formation of initial complexes that are then merged, and by Pan et al. (2019) in that adjacent node similarity has been optimized for disjoint community detection. Modularity optimization as in the Louvain algorithm has been combined with the *WCC* score to create a weighted overlapping community clustering metric to detect overlapping communities (Cohen, Hendler & Rubin, 2016). Bartesaghi, Clemente & Grassi (2023) generalized the clustering coefficient to multiplex networks but without using it for community detection.

Closed triangle counting and *WCC* were combined with node features for community detection in Gao, Sun & Gu (2022). Inuwa-Dutse, Liptrott & Korkontzelos (2021) have developed a method for community detection that, in addition to structural properties from connections between nodes, also exploits textual information from nodes and that is therefore dedicated to social networks with their particular type of node features.

### Enabling large scale community detection

Recently, there has been improvement in the computation time for triangle counting (Huang et al., 2022; Yasar et al., 2020), but it has not been adapted to and optimized for the task of community detection yet.

The *WCC* score has been adapted by Lyu et al. (2020) in their Fox algorithm to enable faster overlapping community detection. This is the approach that LazyFox is based on and we describe it in more detail in the ‘Methods’ section. Also, Prat-Pérez, Dominguez-Sal & Larriba-Pey (2014) suggested a computationally faster estimation of the *WCC* score for runtime improvement that does not require counting all triangles explicitly.

On a different track, the parallel Louvain method with refinement (PLMR) and parallel label propagation for disjoint community detection have been proposed for improved runtime (Staudt & Meyerhenke, 2016). Several heuristics for parallelization of community detection have been suggested (Lu, Halappanavar & Kalyanaraman, 2015). Based on the *WCC* score, a distributed community detection method in Spark has been implemented (Abughofa et al., 2021). While the authors demonstrated an improvement in runtime of the single steps, their focus remained on the problem of dynamic graphs in that edges are added over time and their largest dataset contains only few million nodes.

## Methods

We introduce LazyFox, with its required input data and preprocessing, the employed metric $\widehat {WCC}$, and the performed steps in the algorithm. We describe the parallelization that sets LazyFox apart from Fox (Lyu et al., 2020) and allows LazyFox to scale across multiple CPU cores, and finally the performance measures employed here.

### Input data

Our algorithm LazyFox takes the edge list of an undirected, unweighted graph $G(V,E)$ as input, *V* denoting the nodes and *E* the edges of the graph. We assume this graph to be connected for the theoretical discussions, however, LazyFox will still work on a graph with multiple connected components.

### Preprocessing

LazyFox performs the following preprocessing steps on the input edge list of *G*:

#### Remove multi-edges

In graphs a pair of nodes can be connected *via* multiple edges. Since our research on Fox and LazyFox works on “by-bond” communities, we regard multiple connections between two entities as one. Therefore, we remove such duplicated edges, turning any input multi-graph into a simple graph.

#### Dense node labels

The node identifiers (IDs) in edge lists are not necessarily consecutive due to preprocessing (this is the case in most datasets introduced in the ‘Datasets’ section). We apply a shift to the node IDs to restore their consecutive nature. This allows both Fox and LazyFox to use the node IDs directly as data-structure indices which speeds up computation.

#### Node order

As LazyFox computes communities by gradually improving node memberships, there needs to be a well defined order in which the nodes are being processed. We define this order by sorting the nodes by decreasing clustering coefficient (CC):

(1) $$CC(i) = \left\{ {\matrix{
   {{{{k_i}} \over {{d_i} \cdot ({d_i} - 1)/2}},} & {{\rm{if }\,\,\,}{d_i} > 1,}  \cr 
   {0,} & {{\rm{else}}}  \cr 

 } } \right.$$

${k_i}$ denotes the number of triangles containing node $i$ (equal to the number of edges between two neighbors of node $i$), while ${d_i}$ denotes the degree of node $i$. A higher CC indicates that a node is central in its neighborhood, and we process more central nodes first. Ties are resolved by node degrees in decreasing order. This order ensures that nodes with less connectivity and thus less influence adapt to changes in memberships of more important, connected nodes and not vice versa, resulting in a more coherent community structure at the end of one iteration. If a tie cannot be resolved by CC or node degree, the node ID is used to break the tie.

#### Initial clustering

To initiate the clustering process LazyFox computes a greedy, non-overlapping community decomposition using the above node order. A node that is not yet part of a community is assigned to a new community. Then all its not yet assigned neighbors join this community.

This process allows for self-initialization on any given network. LazyFox derives the initial community count and the initial clustering from the structure of the underlying network. On the other hand, LazyFox is, just like Fox (Lyu et al., 2020), also able to improve upon an existing division into communities by replacing the initial clustering step by inserting those existing divisions. Therefore, known structural properties can be taken into account. This way LazyFox can be used to generate overlapping community structure from partitions, *i.e*., from non-overlapping community structure. See Lyu et al. (2020) for a discussion and examples.

### $\widehat {WCC}$—a metric to optimize for

Lyu and colleagues introduced $\widehat {WCC}$ as an advanced metric to assess the quality of a partition into communities. This metric forms the core of both the Fox and the LazyFox algorithm as they use $\widehat {WCC}$ to decide on the necessary local optimization steps. To provide a better understanding of $\widehat {WCC}$ we will first describe *WCC*.

#### WCC

The weighted clustering coefficient *WCC* as introduced by Prat-Pérez et al. (2012) is a score that rates a community decomposition as the sum of its community ratings. We denote such a rating of a community decomposition $P = \{ {C_1}, \ldots ,{C_k}\}$ as

(2) $$WCC(P) = \sum\limits_{i = 1}^k W CC({C_i})$$

This rating of an individual community ${C_i}$ in Eq. (2) can be again decomposed into how well the individual nodes of that community fit into the community:

(3) $$WCC({C_i}) = \sum\limits_{x \in {C_i}} W CC(x,{C_i})$$

Such a fit of a node $x$ into its community *C* is assessed by the ratio of $t(x,C)$ to $t(x,V)$: The number of triangles that the node forms within its community to the number of triangles that it forms within the whole graph. The assessment also uses $vt(x,V)$, the number of nodes that form triangles with node $x$. Furthermore, $vt(x,V \!\setminus \!\!C)$, the number of nodes outside of the community *C* that form triangles with node $x$, influences this assessment:

(4) $$WCC(x,C) = \left\{ {\matrix{
   {{{t(x,C)} \over {t(x,V)}} \cdot {{vt(x,V)} \over {\mid C \setminus \{ x\} \mid  + vt(x,V \setminus C)}},} & {{\rm{if}}\;t(x,V){\rm{  > 0,}}}  \cr 
   0 & {{\rm{else}}}  \cr 

 } } \right.$$

#### Fast counting of triangles

The counting of triangles for the *WCC* computation is expensive. To accelerate the evaluation of a new community decomposition, Lyu and colleagues replaced the exact counts of $t$ and $vt$ in Eq. (4) by approximations and related quantities, respectively, creating the new, *WCC*-inspired metric $\widehat {WCC}$ (Lyu et al., 2020). First, rather than counting the triangles $t$ a node forms with the nodes of a community *C* and all other nodes, $\widehat {WCC}$ approximates this by using the number of edges instead.

Assuming that the edges inside a community are homogeneously distributed between the nodes, a mean-field approximation can be performed. This delivers the expected triangle count depending on the density $p$ of the community and the degree $deg(x,C)$, which is the count of edges between node $x$ and all nodes within community *C*:

(5) $${\mathbb{E}}[t(x,C)] = \left( \matrix{ deg(x,C) \cr 2 \cr} \right) \cdot p$$

Analogously, one can also define an estimator for the average number of triangles in the whole graph. It uses the global clustering coefficient $cc$ (Watts & Strogatz, 1998), the average of all local clustering coefficients (CC):

(6) $${\mathbb{E}}[t(x,V)] = \left( \matrix{ deg(x,V) \cr 2 \cr} \right) \cdot cc$$

To further decrease the computational cost, the exact values of $vt(x,V)$ and $vt(x,V \!\setminus \!\!C)$ are replaced by their respective upper bound: If all neighbours of $x$ form a triangle with $x$, the upper bound of $vt(x,V)$ is reached—it is exactly $deg(x,V)$. The same holds for all neighbors of the vertices $V \!\setminus \!\!C$, so that the upper bound of $vt(x,V \!\setminus \!\!C)$ is $deg(x,V \!\setminus \!\!C)$. Note that the replacements of $vt$ by upper bounds, however, do not allow to control the deviation of the ratio ${{ vt(x,V)} \over {\mid C \setminus \{ x\} \mid + vt(x,V \setminus C)}}$ that forms the second factor of the *WCC* in Eq. (4).

Thus, a new metric $\widehat {WCC}$ can be derived from the weighted clustering coefficient *WCC*, using Eqs. (5) and (6) and the discussed upper bounds of $vt$:

(7) $$\widehat{WCC}(x,C) = \left\{ {\matrix{
   {{{{\mathbb{E}}[t(x,C)]} \over {{\mathbb{E}}[t(x,V)]}}{{deg(x,V)} \over {\mid C \setminus \{ x\} \mid  + deg(x,V \setminus C)}},} & {{\rm{if }}\,\,\,{\mathbb{E}}[t(x,V)]{\rm{  > 0}},}  \cr 
   {0,} & {{\rm{else}}}  \cr 

 } } \right.$$

Eq. (7) can then be used to replace the usage of $WCC(x,C)$ in Eq. (3), defining $\widehat {WCC}$ for whole communities ${C_i}$

(8) $$\widehat {WCC}({C_i}) = \sum\limits_{x \in {C_i}} {\widehat {WCC}} (x,{C_i})$$

Furthermore, Eq. (8) can replace the usage of $WCC({C_i})$ in Eq. (2), defining $\widehat {WCC}$ for community decompositions $P = \{ {C_1}, \ldots ,{C_k}\}$:

(9) $$\widehat {WCC}(P) = \sum\limits_{i = 1}^k {\widehat {WCC}} ({C_i})$$

Both Fox (Lyu et al., 2020) and LazyFox use $\widehat {WCC}$ as defined in Eq. (9) to compute the optimization steps, as it is faster than the *WCC* metric.

### Node changes

Starting with the initial, non-overlapping community decomposition obtained by the preprocessing step, Fox and LazyFox process the nodes of our graph in multiple iterations. One iteration here is equivalent to computing and applying changes for each node once, described in Algorithms 1 and 2. Before deciding on changes, LazyFox gathers $\widehat {WCC}(P)$, the quality rating of the current decomposition *P*. The LazyFox algorithm then computes a potential *join*- and a potential *leave*-action for the node.

**Table 3:** Comparison of LazyFox Results to Fox Results. Note that LazyFox with a threadcount of 1 is Fox. F1, F1-Score; ONMI-D, ONMI distance, both calculated with the networkit library (Staudt, Sazonovs & Meyerhenke, 2014).

Threadcount	Eu-core		DBLP		LiveJournal	
	F1	ONMI-D	F1	ONMI-D	F1	ONMI-D
1	1.0	0.0	1.0	0.0	1.0	0.0
2	0.99	0.02386	0.99	0.00005	0.99	0.00348
4	0.99	0.03123	0.99	0.00007	0.99	0.00564
8	0.99	0.03005	0.99	0.00008	0.99	0.00684
16	0.99	0.03316	0.99	0.00014	0.99	0.00756
32	0.99	0.05028	0.99	0.00017	0.99	0.00799
64	0.99	0.04884	0.99	0.00027	0.99	0.00837
128	0.99	0.05345	0.99	0.00034	0.99	0.00874
256	0.85	0.31553	0.99	0.00055	0.99	0.00950

#### Joining a community

For the current node $x$, LazyFox finds all communities that $x$ is currently not part of but any of $x$’s neighbors are. Each of these communities ${C_k}$ is then evaluated by creating a decomposition *P′* that differs from the current decomposition *P* by adding $x$ to ${C_k}$ and then computing the, potentially negative, improvement in $\widehat {WCC}(P^\prime)$ compared to $\widehat {WCC}(P)$. If any of these changes yields a positive improvement, we choose the community with the highest increase in $\widehat {WCC}$ as the current *join*-action. This is described in lines two to six in Algorithm 1.

**Algorithm 1:** Fox (Lyu et al., 2020)

1: **for** node $x \in$ Graph **do**
2: bestJoin = undefined
3: **for** community *c* that does not contain *x* and is a neighbor of *x* **do**
4: join = partition if *x* joined *c*
5: update bestJoin if the join partition has a higher $\widehat {WCC}$ score
6: **end for**
7: bestLeave = undefined
8: **for** community *c* that contains *x* **do**
9: leave = partition if *x* left *c*
10: update bestLeave if the leave partition has a higher $\widehat {WCC}$ score
11: **end for**
12: apply bestLeave and bestJoin to the partitioning
13: **end for**

**Algorithm 2:** LazyFox

1: nodeQueue = EmptyList()
2: **for** node x *∈* Graph **do**
3: nodeQueue.insert(x)
4: **if** nodeQueue.size == queueSize **or** x is last node **then**
5: **for** node q *∈* nodeQueue **do**
6: **run** on separate thread
7: bestJoin = undefined
8: **for** community *c* that does not contain *x* and is a neighbor of *x* **do**
9: join = partition if *x* joined *c*
10: update bestJoin if the join partition has a higher $\widehat {WCC}$ score
11: **end for**
12: bestLeave = undefined
13: **for** community *c* that contains *x* **do**
14: leave = partition if *x* left *c*
15: update bestLeave if the leave partition has a higher $\widehat {WCC}$ score
16: **end for**
17: **end run**
18: **end for**
19: collect bestLeaves and bestJoins as nodeChanges from threads
20: apply nodeChanges to the partitioning
21: nodeQueue = EmptyList()
22: **end if**
23: **end for**

#### Leaving a community

Similarly LazyFox checks all communities ${C_k}$ of the current node $x$ and evaluates the gain in $\widehat {WCC}$ if $x$ leaves that community. Therefore, we again form a *P′* per community by removing $x$ from ${C_k}$ and gather the improvement in $\widehat {WCC}$ compared to the old decomposition. If any of these improvements is positive, we again choose the community with the highest increase in $\widehat {WCC}$ as the current *leave*-action. This is described in lines seven to eleven in Algorithm 1.

After determining the best *join*-action and *leave*-action for a node, these are executed yielding a new decomposition as the new *P* before calculating change actions for the next node. This is described in line twelve.

#### Removing degenerated communities

As these node changes allow nodes to leave communities, communities can degenerate to having less than two members. This means that no triangle can be formed with that community anymore. This makes the community un-joinable: A potential joining node $j$ is a node connected to any node in the community *C*. As the community contains only one node $i$, the in-going degree to the community is exactly one. This means that the quantity in Eq. (5) becomes zero $\bigg({\rm as}\; deg(j,C)$ is one, making $\left( \matrix{ deg(j,C) \cr  2 \cr} \right) = \left( \matrix{ 1 \cr  2 \cr} \right) = 0\bigg)$, making $\widehat {WCC}(j,C)$ zero. So joining *C* never improves $\widehat {WCC}$, which means $j$ will not join *C*. This happens both in Fox and LazyFox. While Lyu and colleagues do not explicitly address this in their article, we assume they remove such degenerated communities after each iteration, as they cannot be joined anymore and therefore do not influence the final result. Based on this assumption, we also remove communities with less than two members (meaning they are degenerated) after each iteration. Note that removing a single-node community does not imply removing the node itself.

**Figure 1:**
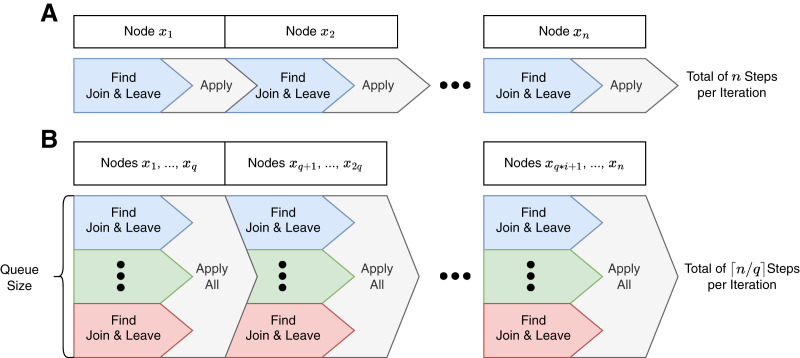
Program control flows of a Fox (A) and LazyFox (B) iteration. Each color denotes a separate thread. While Fox (A) is computed sequentially on one thread, LazyFox (B) can leverage up to queue size many threads that are synchronized by applying their respective node changes. Queue size is denoted as $q$. Note that each Find-Apply-Block in LazyFox contains $q$ nodes, except for the last one, as the total number of nodes of the graph, $n$, might not be a multiple of $q$.

In Fox, the steps of one iteration are sequential and executed on one single thread, visualized in Fig. 1A.

### Parallelism on node change computations

To leverage the advantage of modern multi-core computer systems, LazyFox is capable of parallelizing the computations for the *join* and *leave*-actions for a queue of nodes. Figure 1B visualizes the parallel nature of a LazyFox iteration. Every compute thread fetches a node from the node queue and computes the best actions independently. After the queue is emptied, LazyFox gathers all the computed *join*-actions and *leave*-actions and applies all of them before re-filling the queue with the next set of nodes of the current iteration.

The core process for calculating node changes in LazyFox is the same as in Fox (see Algorithm 2): The lines seven to 16 of Algorithm 2 are the same as the lines three to eleven of Algorithm 1. The difference is the parallel computation of $queueSize$ many nodes in LazyFox (lines one to six) and the bulk apply of node changes in lines 19 to 21.

The parallel computation introduces the first hyper-parameter of LazyFox—the queue size. The parallelization is what sets LazyFox apart from Fox.

### Stopping criteria

While computing and applying the *join*-actions and *leave*-actions, LazyFox updates the $\widehat {WCC}$ of the partition. At the end of each iteration, the current $\widehat {WCC}$ is compared to the $\widehat {WCC}$ of the partition an iteration earlier. If the total relative improvement of $\widehat {WCC}$ is below a $\widehat {WCC}$-threshold—a second hyper-parameter—LazyFox stops. The first iteration of LazyFox is compared to the $\widehat {WCC}$ of the initial node clustering described in the Preprocessing paragraph.

### Post-processing

In LazyFox’ final result, individual communities can not only overlap but also contain each other. The overlap can also lead to duplicated communities. Depending on the use-case, such duplicated or contained communities can be unwanted and have to be removed as part of the post-processing. While we provide an API to include custom post-processing of the final result, LazyFox defaults to not post-process the results.

### Evaluation criteria

LazyFox improves the runtime of the community detection *via* the Fox approach. To evaluate the quality of the results from LazyFox we utilize overlapping normalized mutual information (ONMI) distance^1^ 1OverlappingNMIDistance, https://networkit.github.io/dev-docs/python_api/community.html?highlight=overlappingnmi#networkit.community.OverlappingNMIDistance, retrieved 15.09.2021. and an appropriate F1-Score^2^ 2CoverF1Similarity, https://networkit.github.io/dev-docs/python_api/community.html?highlight=coverf1similarity#networkit.community.CoverF1Similarity, retrieved 15.09.2021., two quality measurements that are widely adopted in the field of overlapping community detection.

#### Overlapping NMI distance

We use a recent variation of the common Normalized Mutual Information to enable its use for overlapping clusterings: Overlapping Normalized Mutual Information (ONMI) (McDaid, Greene & Hurley, 2011). The core idea of the ONMI score is to compare each combination of a community from the LazyFox output with each community in the ground truth. We then select for each output community the closest community in the ground truth leveraging *lack of information* as our distance function. Then the sum over minimal distances is averaged across all output clusters yielding one score, the ONMI distance, ranging from 0 (best) to 1 (worst). We employ its implementation *via* the OverlappingNMIDistance function in Networkit (Staudt, Sazonovs & Meyerhenke, 2014).

#### F1-score for overlapping communities

To enable the use of the common F1-Score for community partitions we use the approach proposed by Epasto, Lattanzi & Paes Leme (2017) and implemented as CoverF1Similarity function in Networkit (Staudt, Sazonovs & Meyerhenke, 2014). Every community is matched to its best ground truth community, and the resulting F1-Scores are then averaged to form the final F1-Score, ranging from 0 (worst) to 1 (best). A single overlapping community *C′* out of the detected partition *P′* is compared with the regular F1-Score to all ground truth communities *C* out of the ground truth partition *P*. The maximum F1-Score is then chosen as the F1-Score for *C′*:

(10) $${F_1}({P^\prime },P) = {1 \over {|{P^\prime }|}}\sum\limits_{{C^\prime } \in {P^\prime }} {\mathop {\max }\limits_{C \in P} } {\rm{ }}{F_1}({C^\prime },C)$$

The F1-Score for comparing a detected community *C′* and a ground truth community *C* is defined *via* the precision $p(C^\prime,C) = { \frac{|C \cap C^\prime|}{|C^\prime|} }$ and recall $r(C^\prime,C) = { {|C \cap C^\prime|} \over{|C|} }$:

(11) $${F_1}(C^\prime,C) = 2 \cdot { \frac{p(C^\prime,C) \cdot r(C^\prime,C)}{p(C^\prime,C) + r(C^\prime,C)}}$$

### Alternative community detection methods

To gain a better understanding of the performance of LazyFox we chose three alternative community detection methods to compare LazyFox’ results with.

#### BigClam

The BigClam algorithm (Yang & Leskovec, 2013) is an overlapping community detection approach based on a cluster affiliation model. Given a network *G*, BigClam estimates the number of communities and creates an affiliation network, optimizing the model with a matrix factorization approach.

#### OSLOM

OSLOM (Lancichinetti et al., 2011) optimizes communities by comparing to network null models. Generally, membership of a vertex $i$ to a community *C* is determined by the number of edges $i$ has with nodes of *C*, and whether there are significantly more edges than expected according to the null model. This algorithm supports hierarchical clustering, where the detected communities can be used to find new communities on a higher hierarchical level.

#### Core expansion

The Core Expansion algorithm (Choumane, Awada & Harkous, 2020) does not use modularity optimization, but instead uses the neighborhood overlap metric. Core Expansion uses nodes with a locally maximal neighborhood overlap score as initial community cores. All other nodes iteratively join their closest and strongest community core, until all nodes are assigned to a community.

In the original Core Expansion algorithm, unassigned nodes can have multiple strongest cores, in which case they are left unassigned. In the adapted cdlib implementation^3^ 3Core Expansion Algorithm, https://cdlib.readthedocs.io/en/latest/reference/cd_algorithms/algs/cdlib.algorithms.core_expansion.html, retrieved 05.01.2023. such nodes join all strongest cores, making this an overlapping community detection algorithm.

## Datasets

To validate both our Fox and LazyFox implementation we collected a set of well-known “by bond” (Ren, Kraut & Kiesler, 2007) networks and ran the algorithms on them to either benchmark their performance or test whether our implementations are capable of dealing with the respective input sizes.

All of our datasets were obtained from the SNAP-datasets collection (Leskovec & Krevl, 2014) (see Table 1 for an overview). The networks are provided in an edge list format. In the following subsections, we present the specific datasets that we used with both their semantic and structural properties.

**Table 1:** Dataset size overview.

Dataset	Short identifier	Node count	Edge count	Average degree
Eu-core	eu	1,005	25,571	25.4
DBLP	dblp	317,080	1,049,866	3.3
LiveJournal	lj	3,997,962	34,681,189	8.7
Friendster	friendster	65,608,366	1,806,067,135	27.5

### Eu-core

Eu-core (https://snap.stanford.edu/data/email-Eucore.txt.gz, retrieved 15.09.2021; Leskovec & Krevl, 2014) is our first and smallest benchmarking dataset. It consists of anonymized email data between members of a large European research institution. An edge exists between two members if at least one email has been sent between these members. The Eu-core graph consists of 1,005 nodes and 25,571 edges. The network is very dense with a diameter of length $7$ and a 90-percentile effective diameter of $2.9$. This can be expected from any cooperation, as people working with each other are likely to communicate.

Each member of the research institution belongs to exactly one of the 42 institution’s departments, which provides the ground truth communities of this dataset.

### DBLP

The Digital Bibliography & Library Project (DBLP) is a computer science publication database provided by the DBLP Organisation. The Library consists of more than 5.4 million publications from different journals and conference articles. The DBLP dataset (https://snap.stanford.edu/data/bigdata/communities/com-dblp.ungraph.txt.gz, retrieved 15.09.2021; Leskovec & Krevl, 2014), the second of our benchmarking datasets, is the co-authorship network calculated for these publications. Each of the 1,049,866 edges in this dataset represents co-authorship on a publication between two of the 317,080 authors.

Each publication venue provides a ground truth community for this dataset, where authors who published in the same venue belong to the same community. If a publication venue contains connected components, each connected component provides another ground truth community. Ground truth communities with less than three nodes were removed.

### LiveJournal

The LiveJournal dataset is derived from the LiveJournal social network. In this social network, users can assign mutual friendship and join user-defined groups. For the dataset (https://snap.stanford.edu/data//bigdata/communities/com-lj.ungraph.txt.gz, retrieved 15.09.2021; Leskovec & Krevl, 2014) the 3,997,962 nodes represent users and the 34,681,189 edges are formed from the friendship relations between these nodes.

Each user-defined group provides a ground truth community for this dataset, where users in the same group belong to the same community. If a user-defined group contains connected components, each connected component provides another ground truth community. Ground truth communities with less than three nodes were removed.

### Friendster

Like the LiveJournal dataset, the Friendster dataset (https://snap.stanford.edu/data//bigdata/communities/com-friendster.ungraph.txt.gz, retrieved 15.09.2021; Leskovec & Krevl, 2014) is also derived from the social network bearing the same name. Yet, the Friendster dataset is an order of magnitude larger bringing in 65,608,366 nodes and 1,806,067,135 edges and 8.7 GB of compressed edges. While this scale makes it unattractive for benchmarking Fox and LazyFox, it serves us as a feasibility test for the algorithm and its implementation on large scale.

## Results

The implementation of the LazyFox algorithm enables us to evaluate the runtime improvements and the impact on overlapping community quality of a multi-threaded approach. Note that running LazyFox with a queue size of one is equivalent to the original Fox algorithm.

During the evaluation, the queue size was always equal to the threadcount. The results of this section were computed with the threadcounts in $\{ 1,2,4,8,16,32,64,128,256\}$ and a fixed $\widehat {WCC}$ threshold of 0.01. The computations were made on an HPE XL225n Gen10 machine with 512 GB RAM, and two AMD EPYC 7742 CPUs with 64 cores each.

### Runtime improvements

The main goal of any multi-threaded approach is to improve the runtime. LazyFox enables the parallel calculation of node changes, speeding up this step. The execution of these changes, however, has to be done sequentially, which is the main non-parallelizable part of the algorithm. The runtime measurements were taken without saving the results of the computation to disk to remove the very volatile I/O operation bottleneck from the benchmarks. The preprocessing steps described in the ‘Methods’ section are part of the measurement.

**Figure 2:**
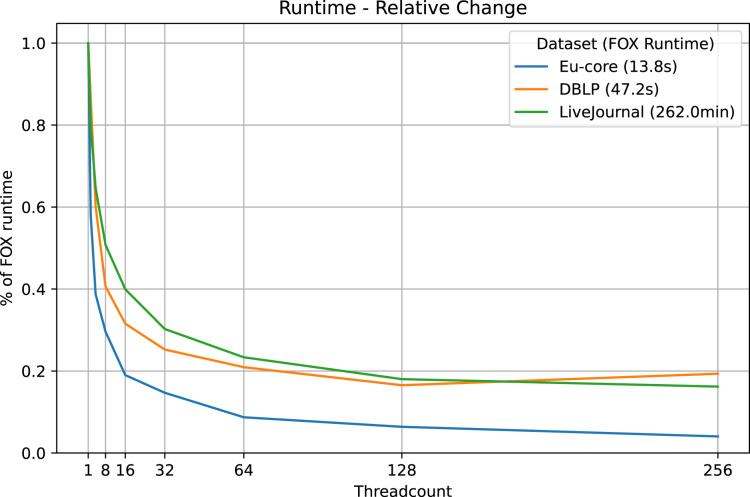
LazyFox runtime comparison. Visualization of the runtimes of LazyFox relative to the non-parallelized runtime (threadcount $1$, Fox algorithm) on the three datasets Eu-core, DBLP, and LiveJournal and different threadcounts using data from Table 2.

We find significant improvements of runtime with the multi-threaded approach on the Eu-core, DBLP and LiveJournal datasets (see Table 2, Fig. 2). Even the use of two threads instead of one reduces the original runtime of the Fox algorithm by about 20%. While extra threads improve the runtime, the improvement rate does not stay the same.

**Table 2:** Runtimes of LazyFox with different threadcounts. The runtimes of LazyFox on three different datasets Eu-core, DBLP, and LiveJournal and different threadcounts relative to the non-parallelized runtime (threadcount 1, Fox algorithm).

Threadcount	Eu-core	DBLP	LiveJournal
1	1.0 (13.8 s)	1.0 (47.2 s)	1.0 (262.0 min)
2	0.58	0.87	0.80
4	0.39	0.60	0.65
8	0.30	0.41	0.51
16	0.19	0.32	0.40
32	0.15	0.25	0.30
64	0.09	0.21	0.23
128	0.06	0.17	0.18
256	0.04	0.19	0.16

The relative runtime improvements in the DBLP and the LiveJournal datasets are roughly the same, despite the fact that the LiveJournal dataset has orders of magnitude more nodes than the DBLP dataset. The original runtime on the Eu-core dataset is reduced massively. With 256 threads LazyFox takes just 4% of the original Fox runtime. However, this extreme improvement is an exception to the overall results, due to the small scale of the Eu-core dataset ( ${\sim}$1,000 nodes spread on $256$ threads). Overall, we observe consistent runtime improvements also for larger scale networks, making the improvements independent of dataset size.

We also see that the runtime of LazyFox on the DBLP dataset gets worse switching from 128 to 256 threads (see Table 2, Fig. 2). This is not caused by an additional iteration that had to be made by LazyFox due to imprecision, as all DBLP runs take exactly seven iterations to converge. Instead, the increase in runtime appears before the first iteration runs, in the run preparations, such as initializing the threads. Thus, the most likely cause for the increased runtime is that the 128 additional thread initializations going from 128 threads in total to 256 threads in total do not yield enough runtime improvement to account for their own initialization time. Taken together, we see a threadcount of 64 or 128 as optimal, being a trade-off between runtime improvement and resource usage for our examined networks.

### Quality impact

The multi-threaded approach of LazyFox does alter the computations. Therefore, the results of LazyFox on the same dataset can differ for different threadcounts. However, we find these changes to be insignificant when comparing to ground truth communities. The following sections compare quality measurements and analysis results of the multi-threaded approaches to the single-threaded approach, and alternative community detection algorithms.

#### Differences in detected communities Fox *vs*. LazyFox

To verify that the difference in community results of LazyFox and Fox are negligible, we compare the results with each other (see Table 3). We use two common metrics to measure overlapping community similarities, F1-Score and ONMI distance (see ‘Methods’). As the computational differences between Fox and LazyFox increase with a higher degree of parallelism, we compare LazyFox with increasing threadcounts.

We find that on sufficiently large datasets, LazyFox results in communities extremely similar to the Fox communities (F1-Score of 0.99, ONMI distance of $\le$0.01). However, we find that for the small Eu-core dataset, a high degree of parallelism does impact the result. While LazyFox with a threadcount of $128$ creates results with 0.99 F1-Score and 0.05345 ONMI distance, the increase to $256$ threads impacts the final result, reducing the F1-Score to 0.85 and increasing the ONMI distance to 0.32. These results suggest that smaller datasets such as Eu-core are more prone to changes caused by parallelization.

#### Ground truth communities and comparison to other community detection approaches

To evaluate the quality of the communities provided by LazyFox we compare them against the ground truth of our datasets. We also evaluate the quality of the results of the Fox algorithm and the algorithms BigClam^4^ 4BigClam Algorithm, https://cdlib.readthedocs.io/en/latest/reference/cd_algorithms/algs/cdlib.algorithms.big_clam.html, retrieved Jan 5, 2023., Core Expansion 9^5^ 5Core Expansion Algorithm, https://cdlib.readthedocs.io/en/latest/reference/cd_algorithms/algs/cdlib.algorithms.core_expansion.html, retrieved Jan 5, 2023., and OSLOM^6^ 6OSLOM Algorithm (beta version 2.4), http://www.oslom.org/, retrieved Jan 5, 2023.. The algorithms BigClam and Core Expansion were run in their default settings provided by the cdlib Python package (version 0.2.6). The OSLOM algorithm was also run with the default settings of the version 2.4.

Note that on the LiveJournal dataset the Core Expansion approach (Core Exp.) and the OSLOM approach did not finish computation after three days. Both runs were aborted. However, OSLOM’s settings offer a ‘–fast’ option, reducing the number of iterations per hierarchy level to one. This enabled us to still retrieve results computed by OSLOM for this dataset and is the only result computed with a deviation from the default settings. Core Expansion offers no such configuration options.

**Figure 3:**
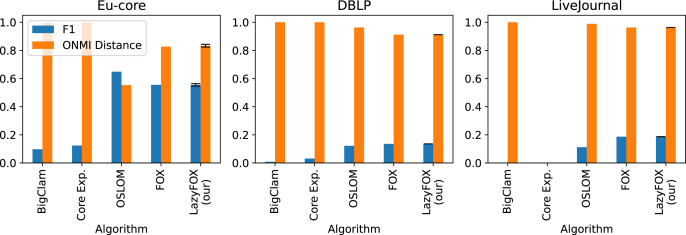
Ground truth comparisons and other methods. Comparison of ground truth communities and detected communities for three different real-world datasets (Table 1) with their ONMI distance (orange bars) and F1-Score (blue). The three community detection methods BigClam, Core Expansion (Core Exp.) and OSLOM are compared with Fox and LazyFox. For our method LazyFox, the orange and blue bars denote the average performance over all eight threadcounts (2, 4, 8, 16, 32, 64, 128, and 256) and the error bars indicate the minimum and maximum of the obtained values, respectively.

Figure 3 displays the performance of the overlapping community algorithms in terms of F1-Score and ONMI distance. We can see that the performance of Fox and LazyFox against ground truth is extremely similar, irrespective of the degree of parallelization of LazyFox. This is especially noteworthy on the Eu-core dataset, as Table 3 shows that the results of Fox and LazyFox differ. This means that while the logic change in LazyFox leads to different communities than Fox, the overall quality of the communities compared to ground truth does not change.

We also see that OSLOM, Fox, and LazyFox outperform the BigClam and the Core Expansion approaches in terms of community quality. While Fox and LazyFox outperform OSLOM on the DBLP and the LiveJournal datasets, OSLOM provides better results on the Eu-core dataset.

Overall we find that despite the much shorter runtime compared to Fox, LazyFox can compete in terms of community quality with other overlapping community detection methods.

#### Community analysis

We also perform a structural analysis of the results obtained by LazyFox. In the best case, the properties measured (such as average community size) should stay the same, regardless of threadcount. Similar properties mean that all threadcounts can be used to draw the same conclusions over community structures within a specific dataset, making the degree of parallelism hyper-parameter used in LazyFox only relevant for run-time improvement, not for result quality.

The most obvious property of community detection is the number of detected communities. The threadcount used has no impact on this number, and for all threadcounts we detected 213 communities for the Eu-core, 76,513 communities for the DBLP and 920,534 communities for the LiveJournal dataset.

Looking at the size of the communities, the maximum and minimum sizes for DBLP and LiveJournal communitites do not change dependent on threadcount. The biggest community in DBLP has 115 members, LiveJournal 574. In the Eu-core dataset the threadcount changes the original maximum size of 135 members by a maximum of plus two. However, the run with 256 threads is an exception, as the maximum size here is decreased by 39. This is another indication that a threadcount close to the node count of the dataset (256 is about one fourth of the Eu-core node count) is not optimal.

Finally, we can investigate the overlap between communities. This is the average number of communities a node belongs to. Again, this value does not change for DBLP or LiveJournal over different threadcounts, staying at 1.19 for DBLP and 1.9 for LiveJournal. The Eu-core dataset’s overlap changes with different threadsizes, starting with an overlap of 5.44 for threadcount equal to one. For a threadcount up to four this stays relatively stable with changes of less than 0.1. However, higher threadcounts change the overlap quite drastically, up to +0.3. Threadcount 256 is the exception here again, changing the overlap from the original 5.44 to 4.22, which is about one community less per node.

From these results we conclude that on sufficiently large datasets, the threadcount does not impact the obtained community structure. The changes only occur with the Eu-core dataset, and get worse with higher threadcounts. This is caused by the small node count in Eu-core. The node count therefore can be used as an indicator to determine the most favorable degree of parallelization. As the Eu-core results already change at two threads instead of one, we would suggest that the node count should at least be 500 times the threadcount to avoid these complications.

### Cluster analysis

While it is worthwhile to investigate the impact of the threadcount on the various metrics, these metrics can also be used to analyze the semantics of the datasets.

LazyFox optimizes triangle count. This means nodes end up in the same group if they are strongly connected with each other. If we speak of people, this means they are socially close. In all our datasets the nodes represent people, connected by bond, such as common work (Eu-core, DBLP) or other social interaction (LiveJournal, Friendster).

### Eu-core

We find 213 communities with an average size of 11. This means that within the research institution, there are about 213 teams with an average team size of 11 people. These teams can already be disbanded, as the data contains e-mails of a long timespan. The high overlap of 5.44 indicates that a single person works on average in 5.44 teams, however, because teams can already be disbanded this is not true for any point in time. It means that an employee works on average in 5.44 teams before leaving the institution or settling for a final research team. This could mean that there are only five or six team changes before reaching a leading position in a team or leaving the company, capping off the team changes.

### DBLP

We find 76,513 communities in the DBLP network with an average size of 4.9 members per community. Semantically these communities represent research groups. Scientists are the nodes, and edges are their co-authorship. Strong connections mean that any author of the group has published at least once with most of the other authors. The average size is reasonable for research groups, and indicates that larger research groups have sub-groups who rarely co-author with each other. Also, there is low overlap between groups, about 1.2, which means each author has on average 1.2 research groups. While one would assume a researcher stays true to their field, the nodes with an overlap above one are likely to be more senior members of the research community, having been part of multiple research groups, or researchers who changed their fields.

### LiveJournal

Another dataset we looked at is LiveJournal, a platform and social network for blogging. Users can befriend each other and join common interest groups. In this dataset we find that the average node has a degree of 17 and an overlap of 1.9. This means that each user has on average 17 friends and belongs to about two friendship groups. We assume these represent real life groups, as we think a mutual friendship circle of average eight people (community size mean) is unlikely to form from social network interactions alone. However, this is an assumption and verifying that would need sensitive data of LiveJournal user accounts to the people behind them and their real life friendships, which we do not have.

### Large scale analysis—Friendster

**Figure 4:**
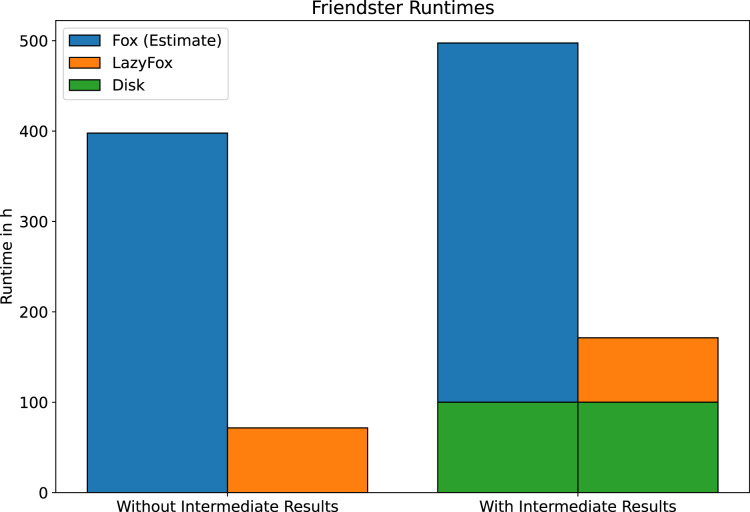
Friendster runtime comparison. The runtime of LazyFox on the Friendster dataset with 256 threads compared to estimates of Fox runtime. Estimates made assuming a speedup comparable to the DBLP and LiveJournal datasets. LazyFox’ parallel computations make Friendster community analysis feasible.

Finally, we would like to present the results of our computations on the Friendster dataset. The enormous scale of this dataset makes an analysis with Fox not feasible in reasonable time. However, due to the runtime improvements achieved with LazyFox this analysis is made possible. We ran LazyFox with 256 threads on the Friendster dataset twice, once with and once without saving the intermediate results to disk. For both runs we use the measured LazyFox runtime to estimate the Fox runtime (see Fig. 4). We do this by assuming a speedup ratio similar to the speedup ratios of the DBLP and LiveJournal datasets ( $0.18$), as we found that the speedup is independent of scale at datasets of sufficient size.

The disk operations of saving the computation results of such a large dataset as Friendster are time consuming: After each iteration about 1.5 GB of clustering results are written to disk. LazyFox converged after eight iterations, running about 171.3 h (~7.1 days) with and 71.6 h (~3.0 days) without saving to disk. It is safe to say that the I/O operations and their preparations take up most of the runtime. This has to be kept in mind when working with large datasets.

Figure 4 illustrates why LazyFox enables community analysis of large scale networks. A runtime of 71.6 h (~3.0 days) is far more feasible than a runtime of almost 400 h (~16.7 days).

After the 8th iteration, LazyFox results in 13,861,732 detected communities, with an average size of nine nodes per community. However, the largest community has 4,721 members. This indicates that Friendster was mostly used for real friend groups where a size of nine people is reasonable, but it also contains closely connected communities of great size. We find that about 92% of all communities have two to 19 members. Communities over 100 members make up only 0.003% of all communities. We assume that these large communities are career networks, institutions or non-human user networks (bots), where a high interconnectedness between members is beneficial to the members, even if they do not know each other personally. We also find that a node belongs on average to 1.92 communities, meaning each user has about two groups of friends.

We find LazyFox highly viable to run analyses on such large datasets, despite the runtime of over a week. However, computing metrics such as F1-Score which compare the results to the graph itself would be challenging due to the sheer size of the graph.

### $\widehat {WCC}$ threshold impact

Another hyper-parameter apart from the threadcount for the LazyFox algorithm is the iteration termination criterion, the $\widehat {WCC}$ threshold. Lyu and colleagues propose a threshold of 0.01 of relative $\widehat {WCC}$ change, meaning that if an iteration decreases the global $\widehat {WCC}$ by less than 1%, the algorithm stops. This threshold was determined by experiments measuring community size and density (compare Table 7 from Lyu et al., 2020), as a lower threshold did not yield significant change in these properties.

**Figure 5:**
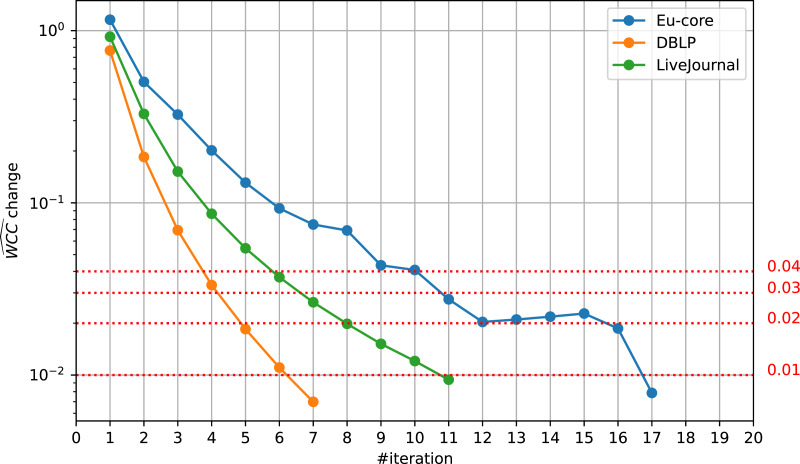
Relative $\widehat {WCC}$ change development. The relative change of $\widehat {WCC}$ at different iterations on different datasets, Eu-core, DBLP, and LiveJournal. By default and also in Lyu et al. (2020), computations stop if the change drops below 0.01. Other possible thresholds (0.02, 0.03, 0.04) are visualized.

However, we find that the threshold’s impact on the algorithm’s runtime is quite extraordinary. As Fig. 5 shows, a slightly higher threshold can save multiple iterations. This has high impact on runtime, as the iterations get slower over time. We find that even the threshold of 0.02 instead of 0.01 yields great runtime improvements for runs on a single thread. For the Eu-core dataset, it saves five iterations, which is equivalent to 39% of the total runtime. For DBLP, we save two, for LiveJournal three iterations, which is equivalent to 33% and 43% of the total runtime, respectively.

These runtime improvements are due to an earlier termination and therefore can still be seen in runs with a higher threadcount. Running LazyFox with 256 threads on the LiveJournal dataset with a $\widehat {WCC}$ threshold of 0.02 still saves 39% of the runtime, compared to the same run with a $\widehat {WCC}$ threshold of 0.01. However, the effectiveness can be dependent on the dataset scale, as the savings for the Eu-core and DBLP datasets at this high threadcount are significantly smaller (3% and 10%, respectively).

**Figure 6:**
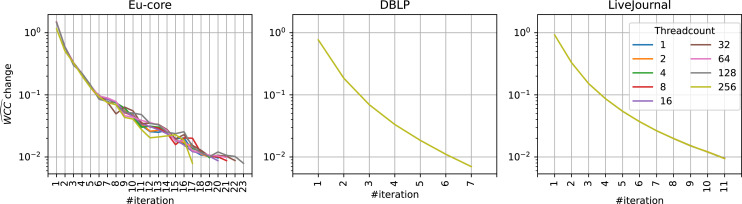
$\widehat {WCC}$ at different threadcounts. $\widehat {WCC}$ development at different threadcounts at different iterations on different datasets, Eu-core, DBLP, and LiveJournal. Threadcount does not influence the $\widehat {WCC}$ development for sufficiently large datasets. Note that in DBLP and LiveJournal datasets the values for each threadcount are exactly the same.

It is important to note that these decreased savings at higher threadcounts are due to the overall decreased total runtime and not due to a different $\widehat {WCC}$ development over time (see Fig. 6). While a higher threadcount can lead to different results and therefore change the $\widehat {WCC}$ scores, this is not the case for large scale datasets. As the community analysis results are unaffected by higher threadcounts (see ‘Quality Impact’ section), so is the $\widehat {WCC}$. The Eu-core dataset here again is an outlier due to the threadcount being close to its node count.

## Discussion

Our proposed parallelization of the algorithm slightly differs in its logic from the original version Fox (Lyu et al., 2020): LazyFox computes first all node changes for a certain batch of nodes (in parallel) and then executes them altogether to generate a new community structure. In contrast, in Fox, computed changes for a single node are applied immediately. However, LazyFox still detects communities which are highly similar to the communities detected by the Fox algorithm. Furthermore, we see that in most cases, our algorithm retrieves the ground truth communities more faithfully than other methods.

These results go along the same lines as the parallelization of *e.g*., the BigClam algorithm (Liu & Chamberlain, 2018). They show once more that many analysis approaches can be parallelized even if the resulting algorithms are not strictly equivalent to their sequential counterparts.

With the runtime improvement provided by the parallelization LazyFox brings overlapping community detection into new areas of application and allows for the analyses of significantly larger and more complex datasets. It reduces the computation time for networks with millions of nodes and billions of edges from weeks to days. For example, LazyFox could not only be used to find community structures in huge social media networks such as Twitter, but also to find complexes or pathways in intricately connected multi-layered molecular network (Liu et al., 2020), or querying global-scale, finely resolved climate (Steinhaeuser, Ganguly & Chawla, 2011), or ecological networks (Bohan et al., 2017). LazyFox is very flexible, the algorithm can be straightforwardly adapted to take directed or weighted graphs into account. We would like to address these extensions in the future, thereby opening even further application areas for overlapping community detection.

LazyFox relies on nodes iteratively joining and leaving communities. Therefore, it could generate empty, disconnected communities, or communities fully contained in other communities. We provide an additional post-processing API to eliminate those types of undesired results. However, note that it can be computationally expensive to perform this post-processing on large graphs, or graphs with many detected communities. Sometimes, communities that are fully contained in other communities are desirable and occur in the ground truth. In these cases, *e.g*., the citation network in SNAP (Yang & Leskovec, 2012), they should not be removed. The post-processing needed is therefore dictated by the research domain which is why post-processing in LazyFox is disabled by default and why detailed analyses on post-processing is not part of our work.

We showed that the value of the $\widehat {WCC}$ threshold hyper-parameter is decisive for the runtime of the algorithm. This is expected as it constitutes the termination criterion of this iterative algorithm, and it is comparable to criteria applied to convergence assessment for other graph-based analyses, such as the PageRank algorithm in NetworKit (Staudt, Sazonovs & Meyerhenke, 2014). Alternatively, limiting the number of performed steps is another solution for termination of iterative algorithms that has been applied previously, *e.g*., in node-centric PageRank computation in iPregel (Capelli et al., 2019). An investigation of the asymptotic behavior, *i.e*., whether the $\widehat {WCC}$ score of LazyFox converges, might be interesting, but we have not experienced problems in the application cases we analyzed. As heuristic solution, we suggest to monitor the change in $\widehat {WCC}$ over the course of the computation to discover problematic cases.

The queue size as second hyper-parameter of LazyFox was set to the available threadcount in our experiments. In principle, LazyFox allows to choose both values independently from one another. However, increasing the threadcount over the queue size does not yield any runtime improvements as the queue size controls the maximum degree of parallelization. Additional threads will then actually slow down the computation as their allocation takes time.

Moreover, the larger the queue size, the more different LazyFox becomes to Fox in theory, and we cannot guarantee that the community detection results remain stable. Overall, we find that the optimal queue size and threadcount should scale with the size of the dataset, but unless very small graphs are analyzed, up to 250 cores can be exploited without strong impact on the results.

## Conclusion

We provide LazyFox, an open-source implementation of an efficient, parallelized algorithm for overlapping community detection. Our implementation and analysis is another positive example for the idea that many graph analysis algorithms can be parallelized, even if not being strictly equivalent to their sequential version. The results show that the in-parallel computed optimization of the $\widehat {WCC}$ metric yields extremely similar results to the sequential computation. This allows the leverage of modern hardware to significantly decrease runtime, enabling the detection of community structures of very large graphs, without losing community quality. The results on the impact of the $\widehat {WCC}$ threshold allow an informed decision on the runtime- $\widehat {WCC}$ score trade-off. With our improvements, we make overlapping community detection achievable for very large graphs with at least tens of million of nodes and a few billion edges in a reasonable amount of time. Thus, we enable this type of graph analysis for novel and complex application domains that have not been previously explored with respect to overlapping community detection.

## Additional Information and Declarations

### Data Availability

The following information was supplied regarding data availability: The code is available at Zenodo: Tim Garrels. (2022). timgarrels/LazyFox: Zenodo DOI Release (v1.2). Zenodo. https://doi.org/10.5281/zenodo.7374484. The employed network data are available from the Stanford Network Analysis Project (https://snap.stanford.edu/data/); Contact Jure Leskovec (jure@cs.stanford.edu): - email-Eu-core dataset: https://snap.stanford.edu/data/email-Eu-core.html - DBLP dataset: https://snap.stanford.edu/data/com-DBLP.html - LiveJournal dataset: https://snap.stanford.edu/data/com-LiveJournal.html - Friendster dataset: https://snap.stanford.edu/data/com-Friendster.html

## References

[ref-1] Abughofa T, Harby AA, Isah H, Zulkernine F (2021). Incremental community detection in distributed dynamic graph.

[ref-2] Ahn Y-Y, Bagrow J, Lehmann S (2010). Link communities reveal multiscale complexity in networks. Nature.

[ref-3] Airoldi EM, Blei DM, Fienberg SE, Xing EP (2008). Mixed membership stochastic blockmodels. Journal of Machine Learning Research.

[ref-4] Barabasi A-L, Gulbahce N, Loscalzo J (2011). Network medicine: a network-based approach to human disease. Nature Reviews Genetics.

[ref-5] Barabasi A-L, Oltvai Z (2004). Network biology: understanding the cell’s functional organization. Nature Reviews Genetics.

[ref-6] Bartesaghi P, Clemente GP, Grassi R (2023). Clustering coefficients as measures of the complex interactions in a directed weighted multilayer network. Physica A: Statistical Mechanics and its Applications.

[ref-7] Basuchowdhuri P, Shekhawat MK, Saha SK (2014). Analysis of product purchase patterns in a co-purchase network.

[ref-8] Blondel VD, Guillaume J-L, Lambiotte R, Lefebvre E (2008). Fast unfolding of communities in large networks. Journal of Statistical Mechanics: Theory and Experiment.

[ref-9] Boccaletti S, Latora V, Moreno Y, Hwang D-U (2006). Complex networks: structure and dynamics. Physics Reports.

[ref-10] Bohan D, Vacher C, Tamaddoni-Nezhad A, Raybould A, Dumbrell A, Woodward G (2017). Next-generation global biomonitoring: large-scale, automated reconstruction of ecological networks. Trends in Ecology & Evolution.

[ref-11] Bu Z, Cao J, Li H, Gao G, Tao H (2017). Gleam: a graph clustering framework based on potential game optimization for large-scale social networks. Knowledge and Information Systems.

[ref-12] Capelli LA, Hu Z, Zakian TA, Brown N, Bull JM (2019). iPregel: vertex-centric programmability vs memory efficiency and performance, why choose?. Parallel Computing.

[ref-13] Cerqueti R, Clemente GP, Grassi R (2021). Systemic risk assessment through high order clustering coefficient. Annals of Operations Research.

[ref-14] Chakraborty T (2015). Leveraging disjoint communities for detecting overlapping community structure. Journal of Statistical Mechanics: Theory and Experiment.

[ref-15] Choumane A, Awada A, Harkous A (2020). Core expansion: a new community detection algorithm based on neighborhood overlap. Social Network Analysis and Mining.

[ref-16] Cohen Y, Hendler D, Rubin A (2016). Node-centric detection of overlapping communities in social networks.

[ref-17] Danon L, Duch J, Diaz-Guilera A, Arenas A (2005). Comparing community structure identification. Journal of Statistical Mechanics: Theory and Experiment.

[ref-18] Dao VL, Bothorel C, Lenca P (2020). Community structure: a comparative evaluation of community detection methods. Network Science.

[ref-19] Ding Z, Zhang X, Sun D, Luo B (2016). Overlapping community detection based on network decomposition. Scientific Reports.

[ref-20] Epasto A, Lattanzi S, Paes Leme R (2017). Ego-splitting framework: from non-overlapping to overlapping clusters.

[ref-21] Evans T, Lambiotte R (2009). Line graphs, link partitions and overlapping communities. Physical Review E: Statistical, Nonlinear, and Soft Matter Physics.

[ref-22] Fortunato S (2010). Community detection in graphs. Physics Reports.

[ref-23] Fortunato S, Newman MEJ (2022). 20 years of network community detection. Nature Physics.

[ref-24] Gao G, Sun A, Gu H (2022). Community detection based on topology and node features in social networks.

[ref-25] Gavin A-C, Aloy P, Grandi P, Krause R, Boesche M, Marzioch M, Rau C, Jensen L, Bastuck S, Dümpelfeld B, Edelmann A, Heurtier M-A, Hoffman V, Hoefert C, Klein K, Hudak M, Michon A-M, Schelder M, Schirle M, Superti-Furga G (2006). Proteome survey reveals modularity of the yeast cell machinery. Nature.

[ref-26] Gopalan PK, Blei DM (2013). Efficient discovery of overlapping communities in massive networks. Proceedings of the National Academy of Sciences of the United States of America.

[ref-27] Gregory S (2010). Finding overlapping communities in networks by label propagation. New Journal of Physics.

[ref-28] Guimerà R, Amaral L (2005). Functional cartography of complex metabolic networks. Nature.

[ref-29] Guimerà R, Mossa S, Turtschi A, Amaral L (2005). The worldwide air transportation network: anomalous centrality, community structure, and cities’ global roles. Proceedings of the National Academy of Sciences of the United States of America.

[ref-30] Hofman JM, Wiggins CH (2008). Bayesian approach to network modularity. Physical Review Letters.

[ref-31] Huang J, Wang H, Fei X, Wang X, Chen W (2022). tc–stream: large-scale graph triangle counting on a single machine using GPUs. IEEE Transactions on Parallel & Distributed Systems.

[ref-32] Inuwa-Dutse I, Liptrott M, Korkontzelos Y (2021). A multilevel clustering technique for community detection. ArXiv e-prints.

[ref-33] Jebabli M, Cherifi H, Cherifi C, Hamouda A (2018). Community detection algorithm evaluation with ground-truth data. Physica A: Statistical Mechanics and Its Applications.

[ref-34] Kelley S, Goldberg M, Magdon-Ismail M, Mertsalov K, Wallace A, Thai MT, Pardalos PM (2012). Defining and discovering communities in social networks. Handbook of Optimization in Complex Networks, Springer Optimization and Its Applications.

[ref-35] Lancichinetti A, Fortunato S (2009). Community detection algorithms: a comparative analysis. Physical Review E: Statistical, Nonlinear, and Soft Matter Physics.

[ref-36] Lancichinetti A, Fortunato S, Kertész J (2009). Detecting the overlapping and hierarchical community structure in complex networks. New Journal of Physics.

[ref-37] Lancichinetti A, Radicchi F, Ramasco JJ, Fortunato S (2011). Finding statistically significant communities in networks. PLOS ONE.

[ref-38] Lee C, Reid F, McDaid A, Hurley N (2010). Detecting highly overlapping community structure by greedy clique expansion. ArXiv e-prints.

[ref-39] Leskovec J, Krevl A (2014). SNAP datasets: stanford large network dataset collection. http://snap.stanford.edu/data.

[ref-40] Liu CHB, Chamberlain BP (2018). Speeding up BigClam implementation on SNAP.

[ref-41] Liu X, Maiorino E, Halu A, Glass K, Prasad RB, Loscalzo J, Gao J, Sharma A (2020). Robustness and lethality in multilayer biological molecular networks. Nature Communications.

[ref-42] Lu H, Halappanavar M, Kalyanaraman A (2015). Parallel heuristics for scalable community detection. Parallel Computing.

[ref-43] Lyu T, Bing L, Zhang Z, Zhang Y (2020). Fox: fast overlapping community detection algorithm in big weighted networks. ACM Transactions on Social Computing.

[ref-44] Ma J, Wang J, Soltan Ghoraie L, Men X, Haibe-Kains B, Dai P (2019). A comparative study of cluster detection algorithms in protein–protein interaction for drug target discovery and drug repurposing. Frontiers in Pharmacology.

[ref-45] Mcauley J, Leskovec J (2014). Discovering social circles in ego networks. ACM Transactions on Knowledge Discovery from Data.

[ref-46] McDaid AF, Greene D, Hurley N (2011). Normalized mutual information to evaluate overlapping community finding algorithms. ArXiv e-prints.

[ref-47] Midoun MA, Wang X, Talhaoui MZ (2021). A pyramidal community detection algorithm based on a generalization of the clustering coefficient. Journal of Ambient Intelligence and Humanized Computing.

[ref-48] Newman M (2006). Modularity and community structure in networks. Proceedings of the National Academy of Sciences of the United States of America.

[ref-49] Omranian S, Angeleska A, Nikoloski Z (2021). Efficient and accurate identification of protein complexes from protein-protein interaction networks based on the clustering coefficient. Computational and Structural Biotechnology Journal.

[ref-50] Palla G, Derényi I, Farkas I, Vicsek T (2005). Uncovering the overlapping community structure of complex networks in nature and society. Nature.

[ref-51] Pan X, Xu G, Wang B, Zhang T (2019). A novel community detection algorithm based on local similarity of clustering coefficient in social networks. IEEE Access.

[ref-52] Ponomarenko A, Pitsoulis L, Shamshetdinov M (2021). Overlapping community detection in networks based on link partitioning and partitioning around medoids. PLOS ONE.

[ref-53] Prat-Pérez A, Dominguez-Sal D, Brunat JM, Larriba-Pey J-L (2012). Shaping communities out of triangles.

[ref-54] Prat-Pérez A, Dominguez-Sal D, Larriba-Pey J-L (2014). High quality, scalable and parallel community detection for large real graphs.

[ref-55] Psorakis I, Roberts S, Ebden M, Sheldon BC (2011). Overlapping community detection using bayesian non-negative matrix factorization. Physical Review E: Statistical, Nonlinear, and Soft Matter Physics.

[ref-56] Raghavan N, Albert R, Kumara S (2007). Near linear time algorithm to detect community structures in large-scale networks. Physical Review E: Statistical, Nonlinear, and Soft Matter Physics.

[ref-57] Regan E, Barabasi A-L (2003). Hierarchical organization in complex networks. Physical Review E: Statistical, Nonlinear, and Soft Matter Physics.

[ref-58] Regan E, Somera A, Mongru D, Oltvai Z (2002). Hierarchical organization of modularity in metabolic networks. Science.

[ref-59] Reid F, McDaid A, Hurley N, Özyer T, Erdem Z, Rokne J, Khoury S (2013). Partitioning breaks communities. Mining Social Networks and Security Informatics.

[ref-60] Ren Y, Kraut R, Kiesler S (2007). Applying common identity and bond theory to design of online communities. Organization Studies.

[ref-61] Saltz M, Prat-Pérez A, Dominguez-Sal D (2015). Distributed community detection with the WCC metric.

[ref-62] Schaub MT, Delvenne J-C, Rosvall M, Lambiotte R (2017). The many facets of community detection in complex networks. Applied Network Science.

[ref-63] Shi C, Cai Y, Fu D, Dong Y, Wu B (2013). A link clustering based overlapping community detection algorithm. Data & Knowledge Engineering.

[ref-64] Song Y, Bressan S, Dobbie G, Hameurlain A, Küng J, Wagner R, Decker H, Lhotska L, Link S (2015). Fast disjoint and overlapping community detection. Transactions on Large-Scale Data- and Knowledge-Centered Systems XVIII: Special Issue on Database- and Expert-Systems Applications.

[ref-65] Souravlas S, Sifaleras A, Katsavounis S (2019). A parallel algorithm for community detection in social networks, based on path analysis and threaded binary trees. IEEE Access.

[ref-66] Souravlas S, Sifaleras A, Tsintogianni M, Katsavounis S (2021). A classification of community detection methods in social networks: a survey. International Journal of General Systems.

[ref-67] Staudt CL, Meyerhenke H (2016). Engineering parallel algorithms for community detection in massive networks. IEEE Transactions on Parallel and Distributed Systems.

[ref-68] Staudt CL, Sazonovs A, Meyerhenke H (2014). NetworKit: a tool suite for large-scale complex network analysis. ArXiv e-prints.

[ref-69] Steinhaeuser K, Ganguly A, Chawla N (2011). Multivariate and multiscale dependence in the global climate system revealed through complex networks. Climate Dynamics.

[ref-70] Vieira VDF, Xavier CR, Evsukoff AG (2020). A comparative study of overlapping community detection methods from the perspective of the structural properties. Applied Network Science.

[ref-71] Wang Y, Chen Q, Yang L, Yang S, He K, Xie X (2021). Overlapping structures detection in protein-protein interaction networks using community detection algorithm based on neighbor clustering coefficient. Frontiers in Genetics.

[ref-72] Wang YJ, Wong GYC (1987). Stochastic blockmodels for directed graphs. Journal of the American Statistical Association.

[ref-73] Watts DJ, Strogatz SH (1998). Collective dynamics of ‘small-world’ networks. Nature.

[ref-74] Xie J, Kelley S, Szymanski BK (2013). Overlapping community detection in networks: the state-of-the-art and comparative study. ACM Computing Surveys.

[ref-75] Xie J, Szymanski BK, Tan P-N, Chawla S, Ho CK, Bailey J (2012). Towards linear time overlapping community detection in social networks. Advances in Knowledge Discovery and Data Mining.

[ref-76] Yang J, Leskovec J (2012). Defining and evaluating network communities based on ground-truth. Knowledge and Information Systems.

[ref-77] Yang J, Leskovec J (2013). Overlapping community detection at scale: a nonnegative matrix factorization approach.

[ref-78] Yasar A, Rajamanickam S, Berry JW, Çatalyürek UV (2020). A block-based triangle counting algorithm on heterogeneous environments. IEEE Transactions on Parallel and Distributed Systems.

